# Toll-Like Receptor 4 Promotes Autonomic Dysfunction, Inflammation and Microglia Activation in the Hypothalamic Paraventricular Nucleus: Role of Endoplasmic Reticulum Stress

**DOI:** 10.1371/journal.pone.0122850

**Published:** 2015-03-26

**Authors:** Gustavo S. Masson, Anand R. Nair, Rahul B. Dange, Pedro Paulo Silva-Soares, Lisete C. Michelini, Joseph Francis

**Affiliations:** 1 Comparative Biomedical Sciences, School of Veterinary Medicine, Louisiana State University, Baton Rouge, Louisiana, United States of America; 2 Department of Physiology and Biophysics, Biomedical Sciences Institute, University of Sao Paulo, Sao Paulo, Brazil; 3 Department of Pharmacology, Fluminense Federal University, Rio De Janeiro, Brazil; SRI International, UNITED STATES

## Abstract

**Background & Purpose:**

Toll-like receptor 4 (TLR4) signaling induces tissue pro-inflammatory cytokine release and endoplasmic reticulum (ER) stress. We examined the role of TLR4 in autonomic dysfunction and the contribution of ER stress.

**Experimental approach:**

Our study included animals divided in 6 experimental groups: rats treated with saline (i.v., 0.9%), LPS (i.v., 10mg/kg), VIPER (i.v., 0.1 mg/kg), or 4-PBA (i.p., 10 mg/kg). Two other groups were pretreated either with VIPER (TLR4 viral inhibitory peptide) LPS + VIPER (i.v., 0.1 mg/kg) or 4-Phenyl butyric acid (4-PBA) LPS + PBA (i.p., 10 mg/kg). Arterial pressure (AP) and heart rate (HR) were measured in conscious Sprague-Dawley rats. AP, HR variability, as well as baroreflex sensitivity (BrS), was determined after LPS or saline treatment for 2 hours. Immunofluorescence staining for NeuN, Ib1a, TLR4 and GRP78 in the hypothalamic paraventricular nucleus (PVN) was performed. TNF-α, TLR4 and GRP78 protein expression in the PVN were evaluated by western blot. Plasma norepinephrine levels were determined by ELISA.

**Key Results:**

Acute LPS treatment increased HR and plasma norepinephrine concentration. It also decreased HR variability and high frequency (HF) components of HR variability, as well BrS. Acute LPS treatment increased TLR4 and TNF-α protein expression in the PVN. These hemodynamic and molecular effects were partially abrogated with TLR4 blocker or ER stress inhibitor pretreatment. In addition, immunofluorescence study showed that TLR4 is co-localized with GRP78in the neurons. Further inhibition of TLR4 or ER stress was able to attenuate the LPS-induced microglia activation.

**Conclusions & Implications:**

TLR4 signaling promotes autonomic dysfunction, inflammation and microglia activation, through neuronal ER stress, in the PVN.

## Introduction

Autonomic dysfunction is implicated in several cardiovascular diseases, such as heart failure [1] and hypertension [2], with prognostic implications. It is characterized by reduced baroreflex sensitivity (BrS), heart rate (HR) variability and cardiac vagal output, as well as increased sympathetic activity. Attenuating autonomic dysfunction is a common theme of many pharmacological agents in treating cardiovascular diseases, using renin-angiotensin system and beta-receptor blockade [3].

Brain inflammation has been identified as a causative pathophysiologic marker in autonomic dysfunction. Several studies from our lab and other groups reported that pro-inflammatory cytokine levels, as tumor necrosis factor-α (TNF-α) and interleukin-1β, are increased in autonomic control areas, such as the paraventricular nucleus (PVN) of the hypothalamus, in experimental models of hypertension and heart failure [4–8]. Acute increase of arterial pressure (AP) and renal sympathetic activity was also reported after pro-inflammatory cytokine injections into the PVN in anesthetized rats [9]. In addition, blockade of pro-inflammatory cytokines or nuclear factor-kappaB (NF-κB), within the PVN, prevented the increase of mean AP and cardiac dysfunction in Angiotensin II induced-hypertension and ischemic cardiomyopathy [4–7]. These findings indicate a major role for inflammatory molecules in cardiovascular diseases.

Microglial activated cells have been suggested as a major cellular source of pro-inflammatory cytokines in experimental models of hypertension and heart failure [10, 11]. Chronic blockade of microglial activation decreased AP and prevented cardiac hypertrophy, as well as reduced pro-inflammatory cytokine expression in the PVN in Angiotensin II induced-hypertension [10]. From a molecular view, role of Toll-like receptor 4 (TLR4) in microglial activation was proposed in several stressor conditions, for instance in ischemia-reperfusion injury [12], chronic alcohol consumption [13] and sepsis [14]. Very few studies have examined the role of TLR4 in cardiovascular disorders. Recently, it was demonstrated that chronic brain TLR4 blockade prevented cardiac remodeling in heart failure [15] and hypertensive rats [16].

In addition to tissue inflammation, persistent endoplasmic reticulum (ER) stress has recently been suggested as a causative agent of hypertension, since the 78kDa glucose regulated protein (GRP78) was over-expressed in the subfornical organ [17] and rostral ventrolateral medulla during a hypertensive response [18]. These studies also identified increased expression of ER stress markers in both autonomic control areas of Angiotensin II induced-hypertensive animals [17, 18]. The cross talk between TLR4 signaling and ER stress is demonstrated by the finding that high fat feeding failed to induce ER stress in TLR4 knock-out mice liver, adipose and skeletal muscle [19]. In addition, Yao et al [20] observed that activation of TLR4 by modified low-density lipoprotein is essential to induce ER stress in macrophages. Considering these data, we hypothesized that TLR4 activation induces acute autonomic dysfunction through ER stress in the PVN. To test our hypothesis, we conducted acute *in vivo* studies in conscious rats, and analyzed cardiovascular and autonomic parameters in response to lipopolysaccharide (LPS), a specific ligand for TLR4. We also performed *ex vivo* experiments and used pharmacological and molecular biology techniques to address the crosstalk between TLR4-induced inflammation and ER stress in the PVN.

## Materials and Methods

### Animals and Experimental Protocol

Animal studies were in accordance with the Guide for the Care and Use of Laboratory Animals as adopted and promulgated by the U.S. National Institutes of Health. Sprague-Dawley rats (2–3 months-old, 250–300 g) were housed in a temperature-controlled room (25±1°C) and maintained on a 12:12 hour light:dark cycle with free access to food and water. Rats were randomized into groups consisting of: LPS (*Sigma-Aldrich*, L3129, LPS from E. coli 0127:B8), a specific ligand for TLR4; LPS + TLR4 blocker (VIPER-TLR4 Viral Inhibitory Peptide); LPS + ER stress inhibitor (PBA—4-Phenyl butyric acid); and controls (saline, VIPER and 4-PBA groups). LPS-treated animals received a bolus injection (*i*.*v*., 10mg/kg). VIPER (*Imgenex*, USA) was injected (*i*.*v*., 0.1 mg/kg) two hours before LPS injection [21]. PBA (*Sigma Aldrich*, USA), an ER stress inhibitor, was administered 16–18 hours (*i*.*p*., 10 mg/kg) before LPS injection. Optimal PBA and VIPER doses were chosen after a preliminary study. Rats in the control group received a saline (*i*.*v*., 0.9%) bolus injection.

### Cardiovascular parameters measurements

Rats (n = 9–10 rats) per group were anesthetized with a ketamine (90 mg/kg) and xylazine (10 mg/kg) mixture (*i*.*p*.) for chronic implantation of catheters in the left femoral artery and vein. The adequacy of anesthesia was monitored by limb withdrawal response to toe pinching. Rats were treated with antibiotics and analgesics and allowed to recover for 3 days. AP and HR were continuously recorded in conscious freely moving rats three days post catheter placement. The arterial catheter was connected to the recording system (transducer, Deltran® II, Utah Medical Products, MidVale, UT, USA + Power Lab system, AD Instruments, Bella Vista NSW, Australia) and 60 min were allowed for the stabilization of cardiovascular parameters (2000 Hz sampling frequency, LabChart 2.0.1). HR was determined from AP pulse interval. After the baseline period, LPS or saline were administrated and cardiovascular parameters were measured for 120 minutes.

### Autonomic parameters measurements

Time series of systolic arterial pressure (SAP) and pulse interval (PI) were generated from each ten minute period after LPS or saline acute treatment. Systolic AP (SAPVar) and HR variability (HRVar) were analyzed in frequency domain. PI is related to the R-R frequency that expresses HRVar. Following linear trend removal, power spectral density was obtained by the Fast Fourier Transformation. Spectral power for low- (LF: 0.20–0.75 Hz) and high (HF: 0.75–4.0 Hz) frequency bands was calculated by means of power spectrum density integration within each frequency bandwidth, using a customized routine (MATLAB 6.0, Mathworks). Coherence between the PI and SAP variability signals was assessed by means of cross-spectral analysis [22].

At the end of 120 minutes of continuous recording, BrS was determined by loading / unloading baroreceptors with graded *iv*. doses of phenylephrine and sodium nitroprusside (0.1–6.4 and 0.2–12.8 μg/kg, respectively, 100 μL bolus injection); subsequent injections were not made until the recorded parameters had returned to pre-injection levels. Mean AP and HR values were measured before (control) and at the peak of each response. Baroreceptor reflex control of HR, determined for each rat, was estimated by the sigmoidal logistic equation fitted to data points, as described previously [21]. The equation linking HR responses to pressure changes was: HR = *P1* + *P2*/[1 + e^*P3*(BP–^ *P4* ^)^], where *P1* = lower HR plateau, *P2* = HR range, *P3* = the curvature coefficient and *P4* = BP_50_ (the value of AP at half of the HR range). The average gain of BrS was calculated as BrS = −(*P2* ×*P3*)/4.Since PBA or VIPER alone did not alter cardiovascular or autonomic function we did not include this group in our molecular characterization.

### Plasma Norepinephrine measurements

Before rats were euthanized, blood was collected from the femoral artery in unanesthetized conditions (n = 9–10 rats). Blood samples were centrifuged at 1800 rpm, 20 minutes, 4°C and the plasma was collected. Norepinephrine (NE) concentration was evaluated through ELISA kit (KA1891; Abnova; USA), according to the manufacturers protocol.

### Immunoblotting

Western blot analysis was performed according to standard protocols as described previously [4, 7]. Since no changes were observed on the cardiovascular and autonomic parameters in the rats treated with TLR4 or ER stress inhibitors alone, we did not apply molecular techniques in these groups. Rat brains (n = 6) were quickly removed and frozen for later procedures. PVN punches were performed in frozen brains sections and homogenized with RIPA lysis buffer. The protein concentration was measured using a bicinchioninic acid protein assay kit (Pierce, USA). Equal amounts of protein (15 μg) were separated by SDS-PAGE on 10% gels, transferred on to PVDF membrane (Immobilon-P, Millipore), and blocked with 1% BSA in TBS-T at room temperature for 60 min. The membranes were incubated with anti-GRP 78 (ab21685; Abcam, 1:1000), anti-TNF-α (sc-8301; Santa Cruz Biotechnology, 1:1000), anti-TLR4 (ab22048; Abcam, 1:500) and anti-actin (sc-1616; Santa Cruz Biotechnology, 1:1000) overnight, at 4 C. The membranes were washed and incubated with anti-rabbit (sc-2004; Santa Cruz Biotechnology, 1:2000) or anti-mouse (sc-2314; Santa Cruz Biotechnology, 1:2000) secondary antibodies for 1 hour at room temperature. Specific bands were detected using an enhanced chemiluminescence kit (GE Life sciences, USA). The bands were visualized using Chemidoc XRS system and Quantity-One software (Bio-Rad) and analyzed using ImageJ software.

### Immunofluorescence

Immunofluorescence staining was performed as described previously [4, 5, 7, 21]. Briefly, sequential hypothalamic coronal sections (30μm, -1.80 to -2.12 caudal to the Bregma) were cut with a cryostat (Leica CM 1850; Nussloch, Germany) and collected in tissue culture wells with 0.1M phosphate buffer (n = 3). Free-floating sections were pretreated with 1% H_2_O_2_-20% ethanol for 30 min, washed with 0.1M phosphate buffer for 25 min and blocked with 2% normal goat serum for 30 min. For the immunofluorescence reaction, the sections were incubated for 48 hours at 4°C, with anti-GRP78 (ab21685, Abcam, 1:400), anti-TLR4 (ab22048 or ab13556, Abcam, 1:250), anti-NeuN (ab104224 Abcam, Ab, 1:1000) and anti-Ib1a (019–19741 Wako, 1:800). Slices were washed with PB 0.1 M for 25 min and incubated for 1 hour at room temperature with Alexa 488 conjugated anti-rabbit (A11034; Invitrogen) or/and Alexa 594 conjugated anti-mouse (A11005; Invitrogen). Four to six slices were placed in each slide and mounted with a coverslip and Vectashield. Negative controls without the primary or secondary antibody were used to determine the background staining. Image analysis was performed with Image ProPlus software. The sections were examined to localize PVN in to 10X and 40X magnification with the same settings for all groups (exposure time-GRP78: 486.88 ms; TLR4: 336.02 ms; NeuN: 236.02 ms; Ib1a: 418.08 ms and sensitivity: ISO16000).

### Statistical Analysis

All data are presented as mean ± SEM and P-values less than 0.05 were considered statistically significant. Statistical analyses were performed using Prism (GraphPad Software, Inc; version 5.0). TWO-way ANOVA (repeated measurements), followed by Bonferroni’s post hoc, was used to analyze cardiovascular and autonomic parameters after LPS or saline infusion (AP, HR, SAPVar and HRVar, as well theirs low and high frequency components). While, for the BrS, plasma NE and all immunoblotting data, One-way ANOVA, with Tukey’s post hoc test, was applied to perform the statistical analyses.

## Results

### TLR4 activation induced autonomic dysfunction

**Fig 1:**
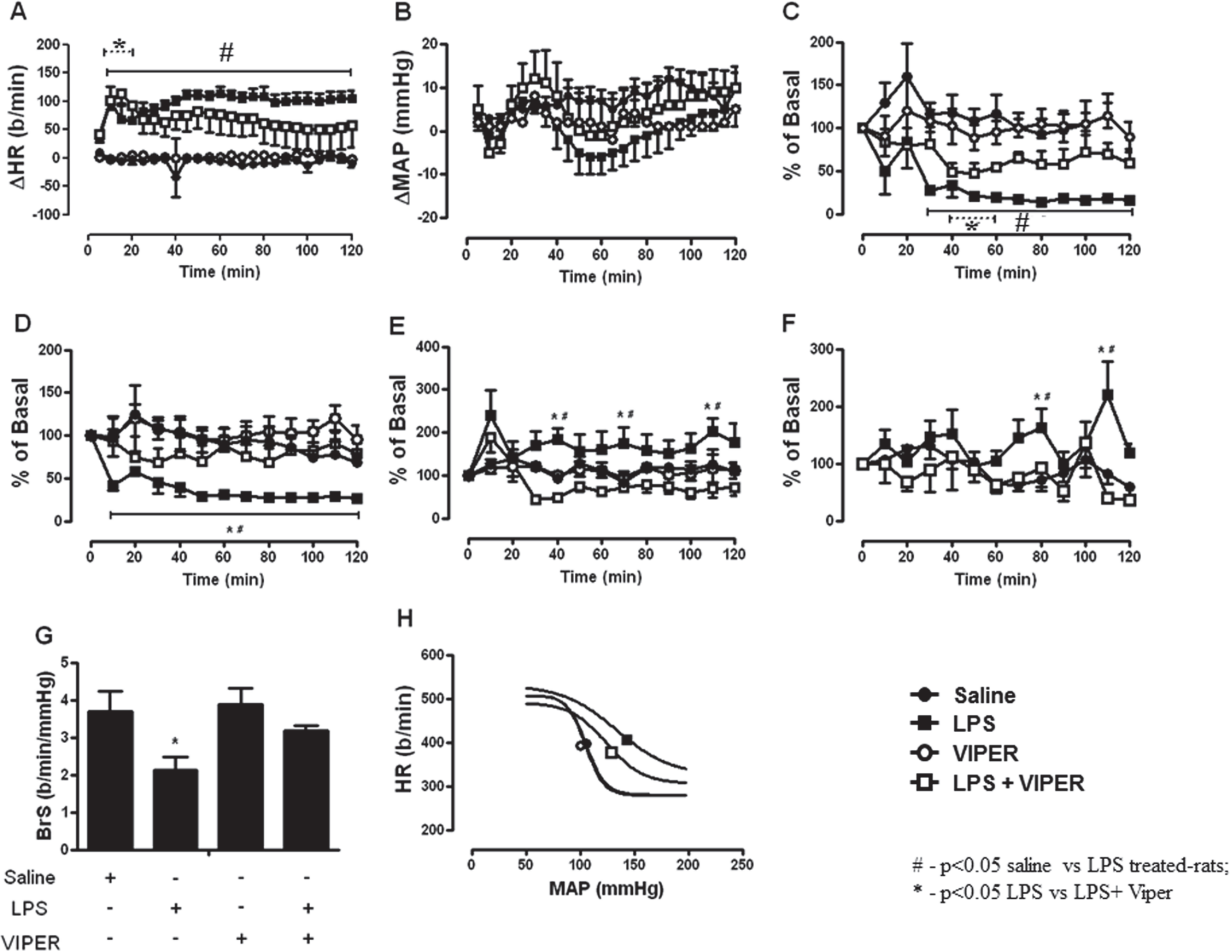
TLR4 signaling induce autonomic dysfunction. (A) Effects of TLR4 blockade (*iv*, 0.1 mg/kg) in LPS-induced changes on heart rate. (B) Effects of TLR4 blockade (*iv*, 0.1 mg/kg) in LPS-induced changes on mean arterial pressure. (C) Effects of TLR4 blockade (*iv*, 0.1 mg/kg) in LPS-induced changes on heart rate variability. (D) Effects of TLR4 blockade (*iv*, 0.1 mg/kg) in LPS-induced changes on high-frequency component of heart rate variability. (E) Effects of TLR4 blockade (*iv*, 0.1 mg/kg) in LPS-induced changes on systolic arterial pressure variability. (F) Effects of TLR4 blockade (*iv*, 0.1 mg/kg) in LPS-induced changes on low-frequency component of systolic arterial pressure variability. (G) Effects of TLR4 blockade (*iv*, 0.1 mg/kg) in LPS-induced changes on baroreflex sensitivity. (H) Effects of TLR4 blockade (*iv*, 0.1 mg/kg) in LPS-induced changes on baroreflex sigmoidal curve. #—p<0.05 saline vs LPS treated-rats; *—p<0.05 LPS vs LPS+ Viper; n = 9–10 rats.

Acute treatment with LPS increased HR from 10 minutes to the end of the protocol, which was inhibited by pretreatment with TLR4 blocker from 60 minutes of the protocol (Fig. 1A). LPS did not significantly change mean AP (Fig. 1B). In addition, acute LPS treatment promptly decreased HRVar and high frequency components of HRVar (cardiac vagal activity marker) from 10 minutes to the end of the experimental protocol. Both effects of LPS treatment were blunted by TLR4 blockade from 60 minutes to the end of the experimental protocol (Fig. 1C and 1D). Although LPS treatment transitorily increased SAPVar at minutes 40, 70 and 110, it increased the low frequency component of SAPVar (peripheral sympathetic marker) at minutes 80 and 110. TLR4 blockade inhibited the increase of SAPVar, as well as its low frequency component, induced by LPS (Fig. 1E and 1F). Consistent with HRVar, we observed that acute LPS treatment decreased BrS and TLR4 blockade inhibited this effect (Fig. 1G). Applying a mathematical model, we plotted the baroreflex curve and we identified the upward and right shift of LPS treated-rats baroreflex curve when compared to saline treated-rats. Interestingly, VIPER pre-treatment caused a downward and left shift of baroreflex curve, which indicates an improvement in baroreflex function (Fig. 1H). Administration of VIPER alone did not change any cardiovascular and autonomic variables measured.

### Role of ER stress in TLR4 induced-autonomic dysfunction

**Fig 2:**
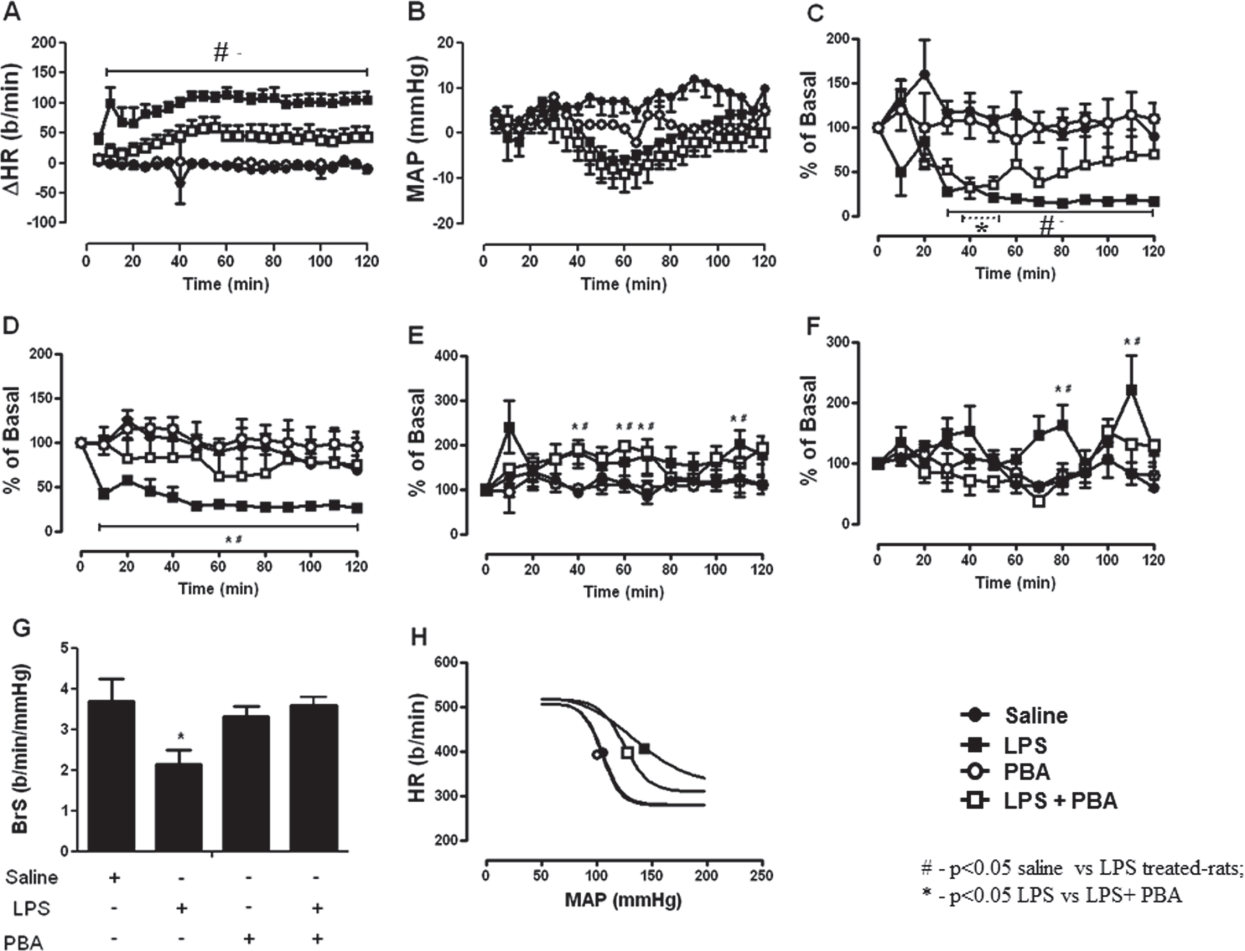
ER stress inhibition prevents autonomic dysfunction induced by TLR4 signaling. (A) Effects of ER stress inhibition (*ip*, 10 mg/kg) in LPS-induced changes on heart rate. (B) Effects of ER stress inhibition (*ip*, 10 mg/kg) in LPS-induced changes on mean arterial pressure. (C) Effects of ER stress inhibition (*ip*, 10 mg/kg) in LPS-induced changes on heart rate variability. (D) Effects of ER stress inhibition (*ip*, 0.1 mg/kg) in LPS-induced changes on high-frequency component of heart rate variability. (E) Effects of ER stress inhibition (*ip*, 10 mg/kg) in LPS-induced changes on systolic arterial pressure variability. (F) Effects of ER stress inhibition (*ip*, 10 mg/kg) in LPS-induced changes on low-frequency component of systolic arterial pressure variability. (G) Effects of ER stress inhibition (*ip*, 10 mg/kg) in LPS-induced changes on baroreflex sensitivity. (H) Effects of ER stress inhibition (*ip*, 10 mg/kg) in LPS-induced changes on baroreflex sigmoidal curve. #—p<0.05 saline vs LPS treated-rats; *—p<0.05 LPS vs LPS+ PBA; n = 9–10 rats.

As demonstrated above, TLR4 ligand LPS, induced acute autonomic dysfunction. Interestingly, ER stress inhibitor PBA pre-treatment was also able to block the HR increase induced by LPS (Fig. 2A). ER stress inhibition blunted the decrease of HRVar evoked by LPS from 60 minutes of experimental protocol (Fig. 2C). We also observed that PBA prevented the decrease of HF component of HRVar induced by LPS during all experimental protocols (Fig. 2D). ER stress inhibition prevented the increase of SAPVar, as well as its low frequency component, induced by LPS (Fig. 2E and 2F). In agreement with HRVar findings, ER stress inhibition prevented acute baroreflex dysfunction evoked by LPS (Fig. 2G). Similarly to TLR4 blockade, PBA treated-rats also exhibited a downward and left shift of baroreflex curve, which suggests an improvement in baroreflex function (Fig. 2H). PBA treatment alone did not affect autonomic or cardiovascular function measurements.

### TLR4 activation increased norepinephrine (NE) release

**Fig 3:**
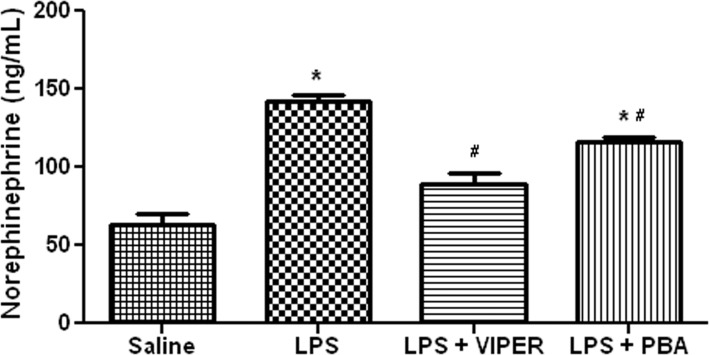
ER stress inhibition prevents raise of plasmatic norepinephrine induced by TLR4 signaling. Effects of ER stress inhibition (*ip*, 10 mg/kg) or TLR4 blocker (*iv*, 0.1 mg/kg) in LPS-induced changes on plasma norepinephrine. *—p<0.05 vs saline treated-rats; #—p<0.05 vs LPS treated-rats; n = 9–10.

Consistent with our HR data, acute LPS treatment increased plasma NE concentration when compared to saline treated animals (136.4±5 vs 52.7±14 ng/mL, p<0.05). TLR4 blockade with VIPER abolished this effect (88.7±7 vs 136.4±5 ng/mL, p<0.05). Interestingly, ER stress inhibitor pre-treatment partially attenuated the increase of plasma NE induced by LPS (125.1±3 vs 136.4±5 ng/mL, p<0.05), since PBA treat rats still exhibited a higher plasma NE compared to saline treated rats (125.1±3 vs 52.7±14 ng/mL, p<0.05) (Fig. 3).

### TLR4 activation by LPS increased ER stress marker GRP78 expression in the PVN

**Fig 4:**
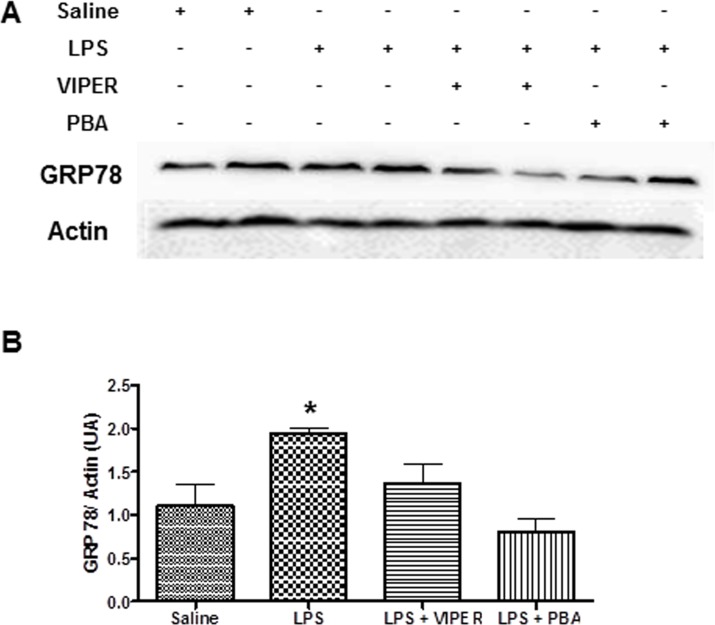
TLR4 signaling induces ER stress in the PVN. (A) Effects of TLR blockade (*iv*, 0.1 mg/kg) or ER stress inhibitor (*ip*, 10 mg/kg) in LPS-induced changes on GRP78 protein expression in the PVN. *—p<0.05 vs others groups; n = 6.

Acute treatment with LPS, when compared to saline treated animals, increased GRP78 protein expression in the PVN (1.97±0.04 vs 1.18±0.02 AU, p<0.05). Pre-treatment with TLR4 blocker (1.43±0.01 vs 1.97±0.04 AU, p<0.05) or ER stress inhibitor (0.81±0.02 vs 1.97±0.04 AU, p<0.05) were able to completely inhibit the increase of GRP78 protein expression induced by LPS in the PVN (Fig. 4A and 4B).

### Inflammation induced by TLR4 activation is suppressed by ER Stress blocker in the PVN

**Fig 5:**
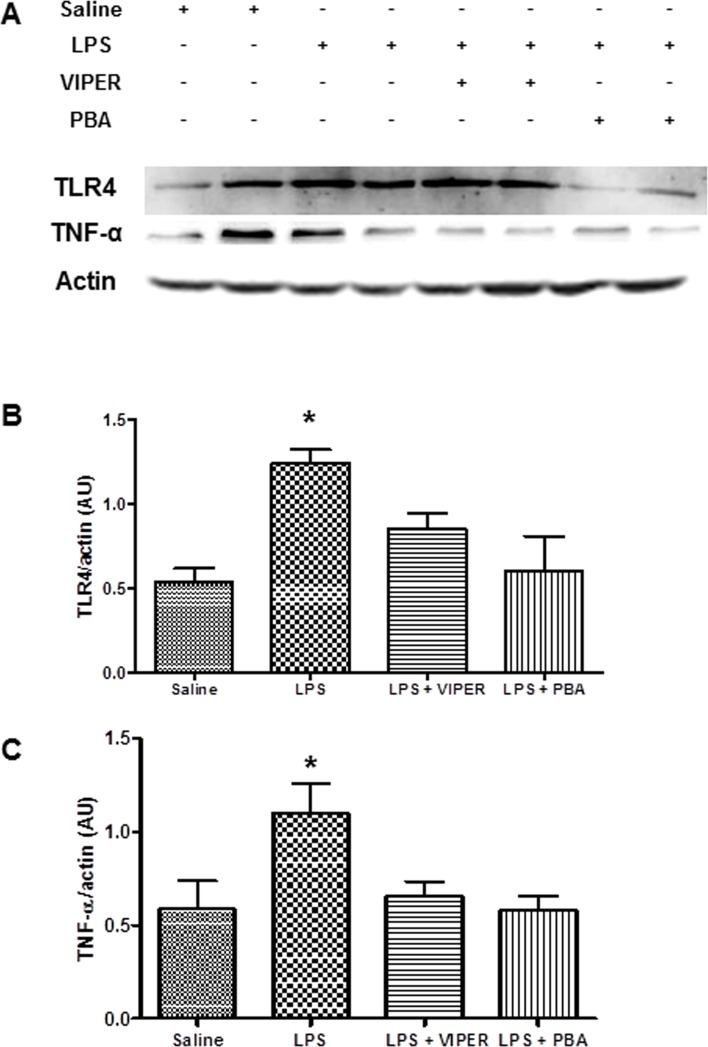
Inflammation induced through TLR4 signaling is dependent to ER stress in the PVN. (A) Representative blots of effects of TLR blockade (*iv*, 0.1 mg/kg) or ER stress inhibitor (*ip*, 10 mg/kg) in LPS-induced changes on TNF-α and TLR4 protein expression in the PVN. (B) Effects of TLR blockade (*iv*, 0.1 mg/kg) or ER stress inhibitor (*ip*, 10 mg/kg) in LPS-induced changes on TNF-α protein expression in the PVN. (C) Effects of TLR blockade (*i*v, 0.1 mg/kg) or ER stress inhibitor (*ip*, 10 mg/kg) in LPS-induced changes on TLR4 protein expression in the PVN. *—p<0.05 vs others groups; n = 6.

Acute treatment with LPS increased TLR4 (1.3±0.08 *vs* 0.53±0.08 AU, p<0.05) (and TNF-α (1.09±0.16 *vs* 0.58±0.15 AU, p<0.05) protein expression in the PVN when compared to the saline group. TLR4 blockade was able to reduce the TLR4 (0.97±0.09 *vs* 1.3±0.08 AU, p<0.05) and TNF-α (0.65±0.08 *vs* 1.09±0.16 AU, p<0.05) protein expression induced by LPS treatment. Interestingly, ER stress inhibition was able to blunt LPS effects on TLR4 (0.60±0.21 *vs* 1.3±0.08 AU, p<0.05) and TNF-α (0.58±0.07 *vs* 1.09±0.16 AU, p<0.05) protein expression (Fig. 5A and 5B).

### TLR4 activation induces neuronal ER Stress in the PVN

**Fig 6:**
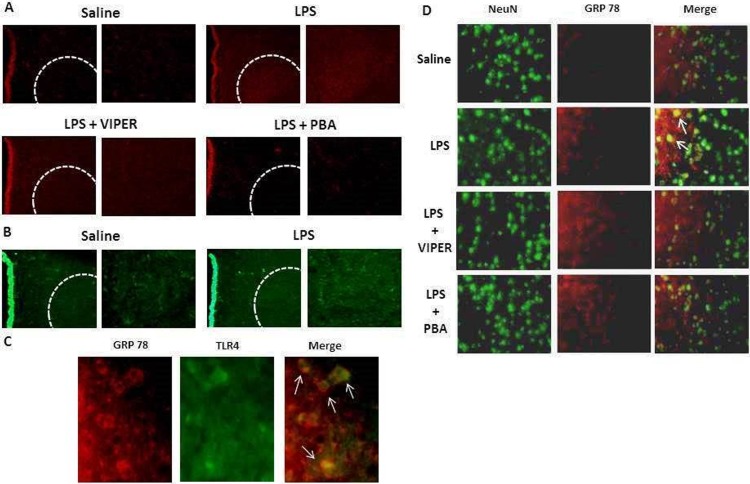
TLR4 and GRP78 immunofluorescence in the PVN. (A) Representative fluorescence microscopy illustrations showing GRP78 single-labeling in the PVN of four experimental groups. (B) Representative fluorescence microscopy illustrations showing TLR4 single-labeling in the PVN of saline or LPS treated rats. (C) Representative fluorescence microscopy illustrations showing TLR4 or GRP78 double-labeling in the PVN of LPS treated rats. Arrows indicate positive TLR4 and GRP78 cells. (D) Representative fluorescence microscopy illustrations showing GRP78 and NeuN double-labeling in the PVN. Arrows indicate positive GRP78 neurons; n = 3.

In order to identify cellular location of ER stress marker GRP78, as well as cellular crosstalk with TLR4, we performed immunofluorescence double-staining in the PVN. Consistent with our immunoblot data, we identified a dramatic increase of GRP78 (Fig. 6A) and TLR4 (Fig. 6B) staining induced by LPS treatment, particularly in the ventral parvocellular area (an autonomic control region). However, VIPER and ER stress inhibitor treatment before LPS administration significantly reduced the increase in GRP78 and TLR4 expression. We observed a co-localization between GRP78 and TLR4 in LPS treated-rats (arrows, Fig. 6C). In addition, GRP78 was co-localized with NeuN, neuronal marker (arrows, Fig. 6D) in LPS treated-rats.

### TLR4 agonist induced neuronal ER stress and microglia activation in the PVN

**Fig 7:**
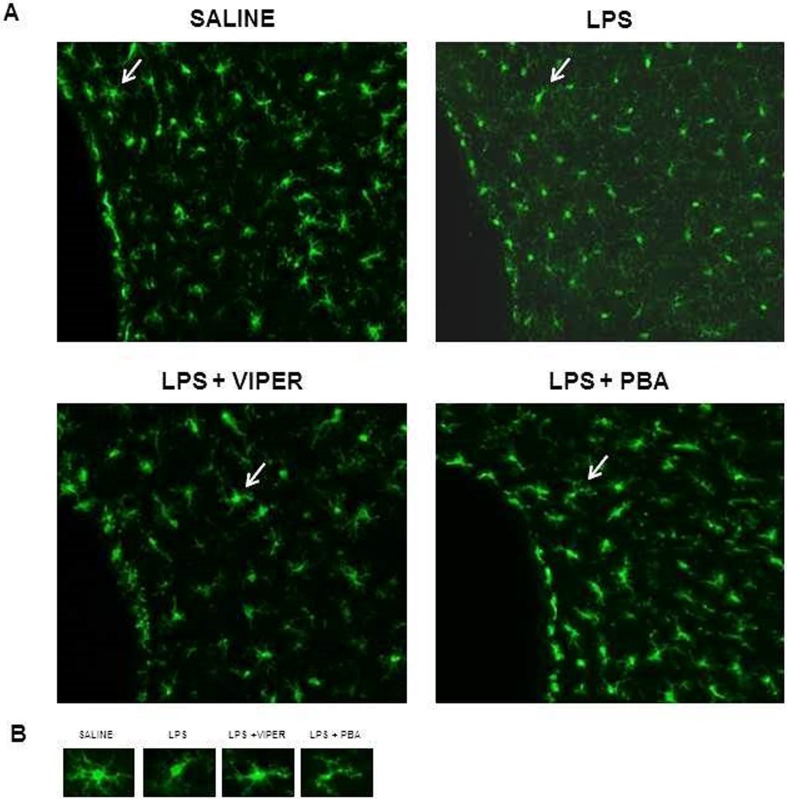
TLR4 activation induced microglia activation through ER stress. (A) Representative fluorescence microscopy illustrations showing Ib1a single-labeling in the PVN of four experimental groups. Arrows indicate amplified microgial cell. (B) Representative fluorescence microscopy illustrations showing microglia in different morphologic levels of activation in four experimental groups; n = 3.

In order to identify a possible source of pro-inflammatory cytokines, we performed immunofluorescence staining for Iba1 (microglia marker) to analyze microglia activation in the PVN. In saline treated-animals, microglia exhibited a higher number of normal morphology cells with numerous long branches and multiple filopodia. Acute LPS treatment induced microglia activation, which was characterized for a dramatic reduction in the cellular branches. TLR4 blocker or ER stress inhibitor pre-treatment were able to decrease microglia activation induced by LPS (Fig. 7A and 7B).

## Discussion

In the present study, we demonstrate for the first time, that acute LPS-induced TLR4 activation, in the PVN, decreases HRVar, cardiac vagal activity and induced baroreflex dysfunction in conscious rats, as well as increases plasma NE. These effects seem to be dependent on ER stress. This is the first report to show that acute LPS (a ligand for TLR4) induces TLR4 and ER stress within the PVN and contributes to autonomic dysfunction. Furthermore, blockade of ER stress attenuates LPS-induced neuroinflammation and TLR4 expression in the PVN, demonstrating a possible role for TLR4-induced brain inflammation and ER stress in autonomic dysfunction. In addition, our immunofluorescence data revealed that acute LPS, a specific TLR4 agonist, induce microglia activation, inflammation and neuronal ER stress in the PVN. These data suggest that TLR4 signaling in the PVN induces autonomic dysfunction through brain inflammation and neuronal ER stress.

Decreased parasympathetic activity and baroreflex function are the hallmark of autonomic dysfunction, which has prognostic value in cardiovascular diseases [1, 2]. The role of brain TLR4 has been recently suggested to modulate cardiac remodeling in heart failure rats [15] and hypertension [16]. Furthermore, chronic LPS treatment has been shown to increase mean AP in healthy rats [23]. In the present study, we observed that acute LPS treatment decreased HRVar, HF component of HRVar (cardiac parasympathetic marker) and BrS. Although SAPVar and its LF components (peripheral sympathetic marker) were increased significantly only at 75 and 115 minutes post-LPS treatment, it evoked an increase of plasma NE, which again indicates an increase in sympathetic activity. The effects induced by LPS were abolished with pretreatment with TLR4 blocker VIPER, indicating a direct role for LPS-induced autonomic dysfunction. Taken together, our data demonstrates, for the first time, that in an acute condition, TLR4 signaling promotes a decrease in cardiac parasympathetic activity and baroreflex function, and increases sympathetic activity.

In addition to autonomic dysfunction induced through TLR4, recent studies have identified a causative role for ER stress in hypertension [17, 18]. In our study, we identified that pre-treatment with an ER stress inhibitor blunted the decrease of HRVar, HF component of HRVar (cardiac parasympathetic marker) and BrS induced by TLR4 activation. In summary, we show for the first time, that TLR4 promotes acute autonomic dysfunction through the induction of ER stress. However, ER stress inhibitor pre-treatment was not able to fully prevent the increase of plasma NE induced by LPS, which demonstrates that LPS induced sympathetic activation is not completely ER stress-dependent.

Recent studies have shown that TLR4 signaling is crucial to development of ER stress [19, 20]. In our study, we identified LPS treatment increases GRP78 protein expression in the PVN, a major autonomic and neuroendocrine region that regulates salt appetite, sympathetic outflow and baroreflex function. LPS-induced GRP78 expression was abolished with TLR4 blockade or ER stress inhibitor indicating a cross talk between TLR4 and ER stress. Taken together with our physiological data, we propose that LPS induced autonomic dysfunction is at least in part modulated by ER stress in the PVN.

TLR4 signaling is a major source of pro-inflammatory cytokines [24], such as TNF-α [25], and blockade of TLR4 in the brain has been shown to prevent cardiac dysfunction in experimental models of heart failure [15] and hypertension [16]. Moreover, chronic LPS treatment was able to increase mean AP and oxidative stress in the rostral ventrolateral medulla [23] indicating a role for TLR4 in sympathetic hyperactivity. Data from our lab demonstrated the role of pro-inflammatory cytokines in the PVN in hypertension and heart failure [4–7]. In our present study, we report that acute LPS treatment increased TNF-α and TLR4 protein expression in the PVN, which were prevented by TLR4 blocker or ER stress inhibitor. These findings indicate that inflammation and ER stress set a positive feedback mechanism, once inflammation elicits ER stress and this cellular condition is able to induce pro-inflammatory cytokines release leading to vicious cycle [26, 27] thereby contributing to autonomic dysfunction. This is supported by our findings that ER stress inhibition attenuated the increase of TNF-α, TLR4 and GRP78. Based on these findings, we suggest that TLR4 signaling-induced ER stress contribute to autonomic dysfunction and brain inflammation.

In addition to our cardiovascular and protein expression data, we performed immunofluorescence staining to characterize the crosstalk between TLR4 signaling and ER stress in the PVN. Acute LPS treatment increased GRP78 and TLR4 protein expression, especially in the ventral parvocellular area, an important cardiovascular autonomic control region in the PVN. Furthermore, in LPS treated rats, TLR4 was co-localized with GRP78, which indicate a possible autocrine mechanism between TLR4 activation and development of ER stress, and these findings are in accordance with previous studies [12–14, 27]. Since we observed GRP78 positive neurons co-localized in the PVN, we can suggest that LPS promotes neuronal ER stress through TLR4 activation. In addition, blockade with TLR4 or ER stress inhibitor decreased co-localization of these markers in the PVN reinforcing a direct crosstalk between TLR4 and ER stress.

Beside the neuronal ER stress induced through TLR4 activation in the PVN, we identified microglia activation, an important pro-inflammatory cytokine source in the PVN. Microglia, a supporting cell in the brain, communicates with neurons and contributes to neuroinflammation in pathological conditions. Here we demonstrate that LPS treatment also increases microglial activation. In saline treated rats, microglia showed numerous long branches which corresponds to an inactivated state. However, LPS modified the cellular morphology to an amoeboid-shape with a decreased number of cellular branches which is related with the pro-inflammatory cytokines secretion [28]. TLR4 blockade or ER stress inhibitor pre-treatment was able to decrease these morphologic modifications in microglia cells induced by LPS in the PVN. Since ER stress inhibitor was able to decrease the microglial activation and PVN cytokines, we can suggest that microglia activation induced through TLR4 is, at least in part, dependent on ER stress. Taken together, our data demonstrated TLR4 activation promotes neuronal inflammation and microglia activation, in the PVN, which contributes to autonomic dysfunction and this effect seems to be, at least in part, ER stress-dependent.

## Conclusion

We performed an acute LPS treatment in conscious rats and utilized pharmacological agents to demonstrate that TLR4 signaling decreases HRVar, cardiac vagal activity and BrS, as well an increase in sympathetic activity through ER stress, neuronal inflammation and microglia activation in the PVN. Our finding suggests that TLR4 signaling pathway and ER stress could be important pharmacological targets for treating some of the autonomic dysfunction in cardiovascular disease.

## Supporting Information

S1 Fig TLR4 immunoflourescence in the PVN. Representative fluorescence microscopy illustrations showing TLR4 single-labeling in the PVN of four experimental groups.
(TIF)
