# Supplementary figures and images for: Toll-Like Receptor 4 Promotes Autonomic Dysfunction, Inflammation and Microglia Activation in the Hypothalamic Paraventricular Nucleus: Role of Endoplasmic Reticulum Stress
Source: PLoS One. 2015 Mar 26;10(3):e0122850. doi: 10.1371/journal.pone.0122850 (PMC4374971; doi:10.1371/journal.pone.0122850)

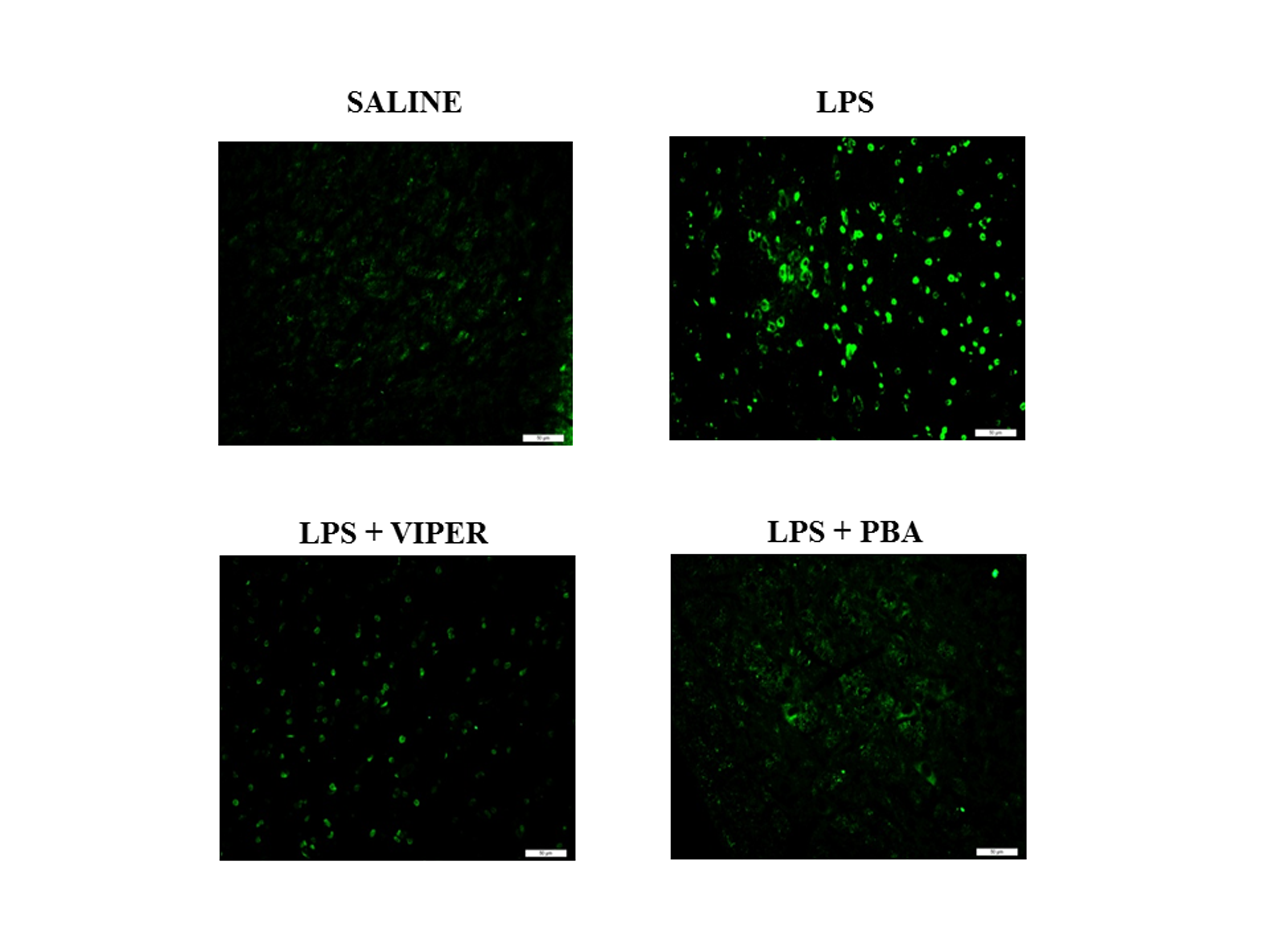

Supplement: S1 Fig — Representative fluorescence microscopy illustrations showing TLR4 single-labeling in the PVN of four experimental groups. (TIF) [file pone.0122850.s001.tif]
